# Predominance of OXA-48 carbapenemase-producing *Klebsiella pneumoniae* strains in tertiary hospital in Sarajevo, Bosnia and Herzegovina

**DOI:** 10.17305/bb.2024.10406

**Published:** 2024-10-01

**Authors:** Amela Dedeić Ljubović, Ðana Granov, Edina Zahirović, Azra Čamdžić, Adis Muhić, Irma Salimović Bešić

**Affiliations:** 1 Unit for Clinical Microbiology, Clinical Centre of the University of Sarajevo, Sarajevo, Bosnia and Herzegovina; 2 Faculty of Health Studies, University of Sarajevo, Sarajevo, Bosnia and Herzegovina; 3 Sarajevo Medical School, Sarajevo School of Science and Technology, Sarajevo, Bosnia and Herzegovina; 4 Department of Clinical Pathology, Cytology and Human Genetics, Clinical Center of the University of Sarajevo, Sarajevo, Bosnia and Herzegovina

**Keywords:** *Klebsiella pneumoniae*, carbapenemases, oxacillinase-48 (OXA-48)

## Abstract

*Klebsiella pneumoniae*, a member of the *Enterobacteriaceae* family, demonstrates an increasing trend of resistance to carbapenems and is a common cause of both hospital- and community-acquired infections. The current study provides insights into the genetic characterization of carbapenem-resistant *K. pneumoniae* (CRKP) isolates circulating during 2022 in a Sarajevo tertiary hospital. Among the 87 CRKP strains analyzed, real-time polymerase chain reaction (rtPCR) results showed that 85 (97.7%) tested positive for the carbapenem resistance gene. The oxacillinase-48 (*OXA-48*) gene was detected in 83 (95.4%) isolates, while the *K. pneumoniae* carbapenemase (*KPC*) and the New Delhi metallo-beta-lactamase (*NDM*) genes were detected in one isolate each. No Verona integron-encoded-metallo-beta-lactamase (*VIM*) or imipenemase-metallo-beta-lactamase 1 (*IMP-1*) genes were found in any of the tested isolates. The multilocus sequence typing (MLST) analysis of sequence types (STs) revealed that ST101, an emerging high-risk clone exhibiting extensive drug resistance, was the most prevalent, whereas ST307 was detected in only one isolate. Phylogenetic analysis of the ten CRKP isolates indicated the presence of three clusters that could constitute an outbreak. A comparison of the results of the utilized phenotypic test (the combined-disk test [CDT]) and rtPCR showed high concordance, suggesting that the phenotypic assay may be useful for the early detection of resistance mechanisms as part of routine susceptibility testing. With the increased affordability of next-generation sequencing (NGS), its application in hospital settings has proven highly beneficial, aiding in the implementation of infection control and prevention measures. Given the significant resistance demonstrated by the CRKP isolates to most tested antibiotics, it is imperative to establish effective methods to restrict the spread of these isolates, as well as to carefully monitor the use of carbapenems in clinical practice.

## Introduction

*Klebsiella pneumoniae*, a member of the *Enterobacteriaceae* family, has shown an increasing trend in antimicrobial resistance and appears as a common cause of hospital- and community-acquired infections [1]. This bacterium tends to spread clonally within healthcare facilities and is particularly adept at causing nosocomial epidemics [2].

Among the different mechanisms of resistance, the acquisition of resistance to carbapenems represents the biggest challenge, given that these antibiotics are often the last therapeutic option for the treatment of infections caused by multidrug-resistant (MDR) gram-negative bacteria [3, 4].

Carbapenem-resistant *K. pneumoniae* (CRKP) can cause serious infections in hospitalized patients, associated with high morbidity and mortality rates. Indeed, that resistance has significantly impacted the treatment of *K. pneumoniae* infections, due to its frequent resistance to commonly used antimicrobial agents used for treating infections caused by gram-negative bacteria [5–7]. Hence, in 2020, it was estimated that CRKP caused 4076 deaths [8].

CRKP primarily arises from the acquisition of carbapenemase genes associated with mobile genetic material, such as plasmids, transposons, and cellular gene cassettes that are transported on integrons [9]. Oxacillinase-48 (OXA-48) is one of the most common carbapenemases found in CRKP strains and has the highest occurrence among carbapenemases in some European Union (EU) countries, including France, Spain, Belgium, and Malta [10].

The 2023 antimicrobial resistance surveillance in Europe, reflecting data from 2021 [11], showed a decrease in carbapenem susceptibility in *K. pneumoniae* and a significantly increasing trend in the EU/European Economic Area (EEA) population-weighted mean percentages for carbapenem resistance from 2017 to 2021, with a proportionally larger increase from 2020 to 2021 compared to the annual changes in previous years. Most often, carbapenem resistance was combined with resistance to several other antimicrobial groups, leading to a limited spectrum of antibiotics available for treating serious infections caused by this pathogen. This underscores the need for continuous, close monitoring, and enhanced initiatives to effectively respond to this growing threat to human health [11].

The nosocomial transmission of carbapenemase-producing *K. pneumoniae* is highly prevalent and noticeable in intensive care units (ICUs), where prolonged use of last-line antibiotics suppresses the normal microbiota, favoring the predominance of resistant strains [12].

Surveillance reports from the Central Asian and Eastern European Surveillance of Antimicrobial Resistance (CAESAR) indicate that the percentage of reported invasive isolates of *K. pneumoniae* from ICU patients in Bosnia and Herzegovina rose from 20% in 2017 to 51% in 2021. Although the resistance rate to carbapenems (imipenem/meropenem) slightly declined in 2021 to 37.1% from 43.5% in 2020, it represents a significant increase from the 10.9% observed in 2017 [11]. This underscores the urgent need to enhance focus on CRKP within our country.

Frequent use of carbapenem antibiotics across various departments of our hospital has led to a year-on-year increase in drug-resistant strains and small outbreaks of hospital infections. The initial occurrences of carbapenemase-producing *K. pneumoniae* at the Clinical Center of the University of Sarajevo were documented in 2017 and 2018.

Primer-specific polymerase chain reaction (PCR) amplification has identified the *bla_OXA-48_* gene as the most prevalent carbapenemase gene [13].

Due to the largely absent or limited molecular characterization of CRKP in our country, this paper aims to present the detection and characterization of carbapenemase-producing *Enterobacteriaceae* in clinical isolates. By evaluating both phenotypic and molecular approaches used in this characterization, the study seeks to provide evidence that may inform effective clinical treatments, as well as strategies for the prevention and control of nosocomial CRKP infections.

## Materials and methods

### Sample collection

A total of 87 non-duplicate isolates of CRKP were collected in 2022. These isolates were identified in various clinical specimens, including swabs from wounds, anus, throat, and nose, as well as from blood, urine, sputum, and aspirates. The specimens were obtained from patients admitted to various clinics at the Clinical Centre of the University of Sarajevo. For preservation, isolates of interest were archived and frozen at −80 ^∘^C in the biobank of the Unit for Clinical Microbiology at the Clinical Center of the University of Sarajevo.

### Phenotypic methods

All samples were cultured on standard (blood agar) and differential culture media, such as chrom agar CPSE (CHROMID CPS Elite Agar, BioMerieux, France), and incubated for 24 h at 37 ^∘^C. Following incubation, isolates were initially identified based on the typical appearance of colonies and standard microbiological procedures. Morphological, cultural, and biochemical analyses were conducted to identify the *K. pneumoniae* isolates, with final identification achieved using the VITEK 2 Compact System (bioMerieux, Marcy l’Etoile, France) equipped with VITEK ID GN cards [13].

Antibiotic susceptibility was tested on Mueller–Hinton agar using the Kirby–Bauer disk diffusion method, adhering to the European Committee on Antimicrobial Susceptibility Testing (EUCAST) standards. The antibiotics tested included ampicillin (10 µg), amoxicillin/clavulanic acid (20/10 µg), piperacillin/tazobactam (30/6 µg), cefazolin (30 µg), cefuroxime (30 µg), ceftriaxone (30 µg), ceftazidime (30 µg), cefepime (30 µg), amikacin (30 µg), gentamicin (30 µg), tobramycin (10 µg), imipenem (10 µg), meropenem (10 µg), ciprofloxacin (5 µg), levofloxacin (5 µg), and trimethoprim–sulfamethoxazole (1.25/23.75 µg).

Additionally, the minimum inhibitory concentration (MIC) determination was performed using the VITEK 2 Compact System with a VITEK AST card, and for colistin, MIC was measured using broth microdilution with MIC-Strip Colistin (Merlin Diagnostika GmbH, Germany). The results were interpreted based on EUCAST breakpoints [14].

Carbapenemase production was detected using a combined-disk test (CDT) containing meropenem and various inhibitors (ROSCO Diagnostica A/S, Denmark), where class A carbapenemases are inhibited by boronic acid and class B by dipicolinic acid and ethylenediaminetetraacetic acid (EDTA). OXA-48-like carbapenemase was identified using temocillin with an MIC >128 mg/L as a phenotypic marker. However, due to its low specificity, this should be verified by additional methods [14].

### Genotypic methods

The detection of MDR genes involved DNA extraction from biological materials followed by real-time PCR (rtPCR) to amplify bacterial DNA. The rtPCR method for identifying MDR genes was based on genome-specific amplification using specific primer/probe sets, commercially available from Sacace Biotechnologies, Como, Italy. A brief overview of the workflow is provided below.

### Bacterial DNA extraction

Bacterial DNA extraction was performed using the QIAcube Connect automated system with the QIAamp DNA Mini Kit (Qiagen, Hilden, Germany), adhering to a spin column protocol that includes lysis, binding, washing, and elution steps for DNA purification from bacterial pellets. Fresh bacterial cultures were grown on MacConkey agar and incubated aerobically overnight at 35 ± 2 ^∘^C. Approximately 5–10 colonies were collected with a 10 µL inoculation loop and suspended in 2 mL of phosphate-buffered saline (PBS) (pH 7.4, Thermo Fisher Scientific, Waltham, MA, USA), by stirring vigorously. The bacteria were then pelleted by centrifugation at 5000 × *g* for 10 min. The pelleted bacteria were processed on the QIAcube Connect shaker to carry out the DNA extraction and purification steps. The elution was performed in two stages. Initially, 100 µL of elution buffer was used, followed by a second elution with 50 µL, selected by the user.

### Multiplex rtPCR

Two multiplex rtPCR assays were performed on 87 isolate samples, identified as carbapenemase-producing *Enterobacteria* (CPE) based on phenotypic characterization. The first assay utilized the MDR *K. pneumoniae* carbapenemase (KPC)/OXA Real-TM kit from Sacace Biotechnologies (Como, Italy) for the rtPCR identification and differentiation of KPC and OXA-carbapenemases in *Enterobacteriaceae* and non-fermenting Gram-negative bacteria (NFGNB). Detection channels were configured as follows: KPC in the 6-carboxyfluorescein (FAM)/Green channel, OXA-48-like enzymes (including OXA-48 and OXA-162) in the 6-carboxy-4′,5′-dichloro-2′,7′-dimethoxyfluorescein (JOE)/hexachloro-fluorescein (HEX)/Yellow channel, and internal control (IC) in the 6-carboxy-X-rhodamine (Rox)/Texas Red/Orange channel.

The rtPCR reaction mixture, totaling 25 µL, comprised 15 µL of master mix (which included 10 µL of PCR-mix-FRT for KPC/OXA-48, 5 µL of RT-PCR-mix-2, and 0.5 µL of polymerase), along with 10 µL of DNA extracted from clinical specimens or controls. Controls included a negative control of extraction (NCE), a negative control of amplification (NCA), and a positive control of PCR (C+). Amplification was carried out on a CFX96 thermocycler (Bio-Rad Laboratories, Hercules, CA, USA) using the following program for plate-type instruments: initial denaturation at 95 ^∘^C for 15 min, followed by five cycles of 95 ^∘^C for 5 s, 60 ^∘^C for 20 s, and 72 ^∘^C for 15 s, then 40 cycles of 95 ^∘^C for 5 s, 60 ^∘^C for 30 s (with fluorescence signal detection), and 72 ^∘^C for 15 s.

The results were interpreted according to the manufacturer’s guidelines. In brief, a result was deemed “positive” for the targeted gene groups if the cycle threshold (Ct) value detected was less than 38 in the respective channels: FAM/Green for the KPC group and JOE/HEX/Yellow for the OXA-48-like carbapenemase group. The results were accepted as significant only if both the positive and negative PCR controls, as well as the negative DNA extraction control, passed correctly, with a Ct value in the ROX/Texas Red/Orange channel (IC) also below 38.

Samples that tested negative in the first rtPCR were subjected to further characterization in a second amplification run. This second multiplex rtPCR test employed the MDR metallo-beta-lactamase (MBL) (Verona integron-encoded-metallo-beta-lactamase [VIM], imipenemase-metallo-beta-lactamase [IMP], and New Delhi metallo-beta-lactamase [NDM]) Real-TM PCR kit (Sacace Biotechnologies, Como, Italy), designed to detect and differentiate the MDR genes. The VIM group was identified in the FAM/Green channel, the IMP group in the JOE/HEX/Yellow channel, and the NDM group in the cyanine 5 (Cy5)/Red channel, while the IC was again detected in the Rox/Texas Red/Orange channel.

The setup for the second rtPCR was similar to the first, with modifications only in the specific primers/probe sets used for the VIM, IMP, and NDM targets. The amplification program was conducted under the same conditions as the initial run. Results were considered “positive” if the Ct values for all targets, including the IC, were below 38.

### Whole genome sequencing

A portion of the extracted bacterial DNA from ten samples was initially quantified using the Qubit dsDNA BR Assay Kit (Invitrogen/Thermo Fisher Scientific, Karlsruhe, Germany). An input of 1 ng per sample was utilized for library preparation. The sequencing libraries were prepared using the Nextera XT DNA Library Prep Kit and IDT for Illumina Nextera DNA UD Indexes (set C; both from Illumina, San Diego, CA, USA). A second DNA quantification was conducted using the Qubit HS DNA Kit, and 1.4 pM of the normalized libraries were loaded onto the flow cell. Whole genome sequencing was performed on the MiniSeq platform (Illumina, San Diego, CA, USA) using v2 chemistry for paired-end reads, in accordance with the manufacturer’s instructions. The raw data (fastq files) were further analyzed.

### Genome profiling and phylogenetic analysis

Genome profiling and phylogenetic analysis were performed using services available at the Center for Genomic Epidemiology (https://www.genomicepidemiology.org/services/), adhering to the default settings.

Sequence types (STs) were determined from the sets of reads utilizing the multilocus sequence typing (MLST) database, employing software version 2.0.9 (released on May 11, 2022) and the database version from June 19, 2023 [15–17].

Identification of antimicrobial resistance genes (ARGs) was performed using ResFinder (new version), employing software version 4.4.1 (released on August 22, 2023) and a database updated on April 12, 2023. Plasmid replicons were identified with PlasmidFinder, using software version 2.0.1 (from July 01, 2020) and database version from January 18, 2023 [16, 18, 19]. Phylogenetic trees were constructed from paired-end reads (fastq files) utilizing NDtree version 1.2 [20–22].

The reference sequences used in these analyses were sourced from the respective databases. The phylogenetic trees were visualized using Unipro UGENE [23]. Additionally, distance matrices generated by NDtree software were examined to assess the potential grouping of outbreak strains based on specified single nucleotide polymorphism (SNP) distances.

### Ethical statement

The study was conducted in compliance with the principles outlined in the Declaration of Helsinki and received approval from the Ministry of Science, Higher Education, and Youth of Sarajevo Canton (Approval No. SVP-27-02-35-35139/22.15T) on September 28, 2022. As the research was performed on bacterial cultures, obtaining informed consent from subjects was not necessary. Nevertheless, all data related to the subjects were encrypted to ensure their anonymity and privacy throughout the study.

## Results

Out of a total of 87 CRKP isolates from the Clinical Center of the University of Sarajevo, the majority were obtained from wound swabs (37.9%), followed by anal swabs (14.9%), blood (11.5%), and urine specimens (10.3%). The remaining isolates (from 2.3% to 8% of cases) were obtained from various other samples (Table 1). CRKP isolates were most frequently isolated from surgical units (48.2%), followed by internal medicine units (29.9%), and ICUs (21.9%) (Table 1).

**Table 1:** Distribution of CRKP isolates by specimen type and hospital departments

**Specimen**	**Surgical units**	**ICU**	**Internal units**	**Total**
Anal swab	5 (5.7)	6 (6.9)	2 (2.3)	13 (14.9)
Secretions	2 (2.3)	0 (0)	1 (1.1)	3 (3.4)
Wound swab	23 (26.4)	5 (5.7)	5 (5.7)	33 (37.9)
Throat swab	1 (1.1)	1 (1.1)	2 (2.3)	4 (4.6)
Tracheal aspirate	2 (2.3)	3 (3.4)	2 (2.3)	7 (8)
Blood	3 (3.4)	1 (1.1)	6 (6.9)	10 (11.5)
Hospital samples	1 (1.1)	2 (2.3)	0 (0)	3 (3.4)
Sputum	1 (1.1)	0 (0)	2 (2.3)	3 (3.4)
Urine	3 (3.4)	0 (0)	6 (6.9)	9 (10.3)
Tip of a drain and CVC	1 (1.1)	1 (1.1)	0 (0)	2 (2.3)
Total	42 (48.2)	19 (21.9)	26 (29.9)	87 (100)

Values are presented as numbers and percentages (%). CRKP: Carbapenem-resistant *Klebsiella pneumoniae*; ICU: Intensive care unit; CVC: Central venous catheter.

Clinical isolates exhibited full resistance to several antibiotics including cefepime, ceftazidime, cefotaxime, ciprofloxacin, gentamicin, levofloxacin, and piperacillin/tazobactam. The MIC for imipenem and meropenem were > 8 µg/mL. Additionally, 3 out of 67 isolates (4.5%) demonstrated resistance to colistin with a MIC of 16 µg/mL. Sensitivity was observed only to amikacin (36 out of 67 isolates, 53.8%) and trimethoprim/sulfamethoxazole (23 out of 67 isolates, 34.4%) (Table 2).

**Table 2:** Antimicrobial susceptibility pattern of CRKP isolates

**Antimicrobials**	**Resistant**	**Intermediate**	**Susceptible**
Amoxicillin/clavulanic acid	67 (100)	0 (0)	0 (0)
Cefazolin	67 (100)	0 (0)	0 (0)
Cefuroxime	67 (100)	0 (0)	0 (0)
Ceftazidime	67 (100)	0 (0)	0 (0)
Cefepime	67 (100)	0 (0)	0 (0)
Gentamicin	67 (100)	0 (0)	0 (0)
Amikacin	9 (13.4)	22 (32.8)	36 (53.8)
Meropenem	67 (100)	0 (0)	0 (0)
Imipenem	67 (100)	0 (0)	0 (0)
Ciprofloxacin	67 (100)	0 (0)	0 (0)
Levofloxacin	67 (100)	0 (0)	0 (0)
Trimethoprim/sulfometoxazole	44 (65.6)	0 (0)	23 (34.4)
Piperacillin/tazobactam	67 (100)	0 (0)	0 (0)
Colistin	3 (4.5)	0 (0)	64 (95.5)

Values are presented as numbers and percentages (%). CRKP: Carbapenem-resistant *Klebsiella pneumoniae*.

The CDT conducted on 85 isolates revealed no variation in the inhibition zones between meropenem disks alone and those combined with inhibitors. However, the inhibition zone diameter for the temocillin disk was less than 11 mm, indicating the presence of the OXA-48 phenotype (Table 3). One isolate exhibited this temocillin zone diameter characteristic of OXA-48 and also showed a synergistic effect, an increase in zone diameter when 10 µg of meropenem was combined with phenylboronic acid, indicative of the KPC phenotype. Conversely, one isolate showed no response in the CDT.

**Table 3:** Resistance genes of CRKP isolates detected by rtPCR and CDT

**Resistance gene**	**rtPCR test**	**CDT OXA-48**	**CDT KPC**	**CDT Negative**	**CDT total**
*NDM*	1 (1.1)	1 (1.1)	0 (0.0)	0 (0.0)	1 (1.1)
*KPC*	1 (1.1)	0 (0.0)	1 (1.1)	0 (0.0)	1 (1.1)
*OXA-48*	83 (95.5)	83 (95.5)	0 (0.0)	0 (0.0)	83 (95.5)
No genes detected	2 (2.2)	1 (1.1)	0 (0.0)	1 (1.1)	2 (2.2)
Total	87 (100.0)	85 (100.0)	1 (1.1)	1 (1.1)	87 (100.0)

Values are presented as numbers and percentages (%). All isolates were tested for the *VIM* and *IMP-1* genes. CRKP: Carbapenem-resistant *Klebsiella pneumoniae*; rtPCR: Real-time polymerase chain reaction; CDT: Combined-disk test; *OXA-48*: Oxacillinase-48; *KPC*: *Klebsiella pneumoniae* carbapenemase; *NDM*: New Delhi metallo-beta-lactamase; *VIM*: Verona integron-encoded-metallo-beta-lactamase; *IMP-1*: Imipenemase-metallo-beta-lactamase 1.

Out of the 87 isolates tested, rtPCR identified 85 (97.7%) as positive for carbapenem resistance genes. Specifically, the *OXA-48* gene was detected in 83 (95.4%) isolates, while *KPC* and *NDM* were detected in one isolate each. No isolates tested positive for the *VIM* or *IMP-1* genes. Two isolates were negative in both rtPCR assays (Table 3).

A comparison between the CDT and rtPCR results showed one isolate negative in both tests. Another isolate tested positive in the CDT for the *KPC* gene and was confirmed by rtPCR. Interestingly, one isolate demonstrated positive results for *OXA-48* in the CDT but showed no genes detected by rtPCR, and another tested positive for *OXA-48* via CDT while rtPCR identified the *NDM* gene (Table 3).

MLST analysis revealed the presence of two different sequence types (STs) among the ten CRKP isolates (BioProject accession number PRJNA 108556). ST101 was the most prevalent, found in 90% (9 out of 10) of the isolates. ST307 was detected in only one isolate (Table 4).

**Table 4:** Distribution of antibiotic resistance genes and plasmid replicons of ten CRKP isolates

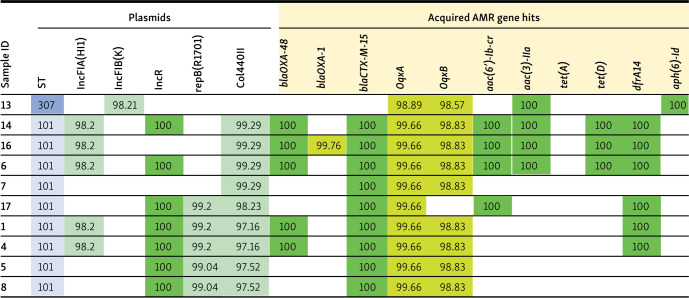

The blue color indicates the most common sequence type. The dark green color signifies a perfect match for a given gene, where the percentage of identity is 100% and the sequence in the genome database covers the entire length of the resistance gene. The light green color indicates a warning due to a non-perfect match, where the percentage of identity is less than 100%, and the HSP length equals the resistance gene length. ST refers to the output from the MLST. Plasmid represents the results obtained from PlasmidFinder. Acquired AMR gene hits and chromosomal mutations mediating AMR denote the results from the updated version of ResFinder. CRKP: Carbapenem-resistant *Klebsiella pneumoniae*; ST: Sequence type; HSP: High-Scoring Segment Pair; MLST: Multilocus sequence typing; AMR: antimicrobial resistance; *OXA-48*: Oxacillinase-48.

As detailed in Table 4, the presence of a dark green color signifies a complete match (100% similarity) between a particular gene and its corresponding sequence in the genome database, encompassing the entire gene length. A light green color indicates a nonperfect match, where the percentage of identity is below 100%, but the High-Scoring Segment Pair (HSP) length is equal to the gene length.

Among the ten isolates, five types of plasmid replicons were identified. The most common replicon type, Col440II, was present in 90% (9 out of 10) of the isolates, followed by IncR, which was found in 70% (7 out of 10). IncFIA(HI1) and repB(R1701) were each identified in 50% (5 out of 10) of the isolates. The remaining plasmid replicon, IncFIB(K), was discovered in the sole ST307 isolate (10%). The prevailing replicon combination was IncR, repB(R1701), and Col440II, accounting for 30% (3 out of 10) of the sequenced isolates (Table 4).

Different acquired antimicrobial resistance genes were identified in ten CPKP isolates, as detailed in Table 4. All ST101 isolates (9 out of 10, representing 90.0% of the sequenced samples) carried the bla*_CTX-M-15_* gene, with five of those also carrying *bla_OXA-48_*. One ST101 sample (ID 16) harbored both *bla_OXA-1_* and *bla_OXA-48_* genes, and was found to carry *OqxA*, *OqxB*, *aac(6’)-Ib-cr*, *aac(3)-IIa*, and *dfrA14* genes. Carbapenemase-producing genes were not detected in the ST307 isolate. However, other acquired antimicrobial resistance gene hits identified in ST307 included *OqxA*, *OqxB*, *aac(3)-IIa*, and *aph(6)-Id*. Detailed profiles of the acquired antimicrobial resistance genes found in the sequenced isolates are presented in Table 4.

**Figure 1.**
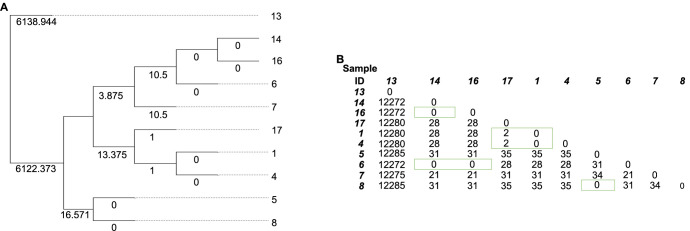
**The phylogenetic tree and distance matrices of ten CRKP isolates**. (A) Displaying the NDtree phylogeny output; (B) Showcasing a symmetrical distance matrix to the diagonal. Green boxes highlight isolates that could constitute an outbreak, grouping together under a cutoff of ten SNPs. CRKP: Carbapenem-resistant *Klebsiella pneumoniae*; SNPs: Single nucleotide polymorphisms.

Phylogenetic analysis of the ten CRKP isolates revealed the presence of three distinct clusters of ST101, which could potentially indicate an outbreak, given a cutoff of ten SNPs (Figure 1A).

According to the distance matrices (Figure 1B), these clusters were constituted of the following isolates/sample IDs: cluster I included IDs 6, 14, and 16; cluster II included IDs 1, 4, and 17; and cluster III included IDs 5 and 8.

## Discussion

*K. pneumoniae*, a common type of gram-negative bacteria, is responsible for infections acquired in healthcare settings, specifically in the bloodstream, urinary system, and respiratory tract. The extensive utilization of carbapenems in medical settings has resulted in a rise in the prevalence of infections caused by strains resistant to these antibiotics, known CRKP.

According to the 2023 antimicrobial resistance surveillance in Europe covering data from 2021, carbapenem resistance in *K. pneumoniae* was generally low in the northern and western regions of the WHO European Region, with 14 of 45 countries (31%) reporting antimicrobial resistance percentages below 1%. In contrast, 15 countries (33%) reported resistance rates of 25% or higher, among which eight countries (18% of the total countries), namely, Belarus, Georgia, Greece, Moldova, Romania, Russia, Serbia, and Ukraine, reported antimicrobial resistance rates of 50% or higher [11].

Surveillance reports from CAESAR showed that in Bosnia and Herzegovina, the resistance rates of invasive *K. pneumoniae* isolates to carbapenems (imipenem/meropenem) were 10.9% in 2017, 18.4% in 2018, 41.7% in 2019, 43.5% in 2020, and 37.1% in 2021 [11].

Research has identified two primary mechanisms contributing to CRKP drug resistance. CRKP becomes resistant to cephalosporins and monobactams through the production of AmpC enzymes or extended-spectrum β-lactamases (ESBLs), combined with overexpression of efflux pump systems, mutations in outer membrane proteins, or alterations in penicillin-binding proteins. The expression of carbapenemases further enhances resistance, making CRKP resistant to nearly all β-lactam antibiotics, including carbapenems [24].

However, there remains a limited understanding of the genetic aspects involved in the initial colonization and subsequent spread of these isolates. The comprehensive genome analysis of individual isolates has facilitated a more accurate understanding of the colonizing strains and their genetic attributes, revealing connections among the isolates. This information is crucial for advising on preventive and management strategies to limit the dissemination of resistance determinants in the future.

In our study, the 87 CRKP strains were predominantly isolated from wound swabs and surgical units. Wound infections are primarily associated with prolonged hospitalization, which increases the risk of acquiring other drug-resistant organisms through medical equipment and the hospital environment [25].

All CRKP strains exhibited complete resistance to most antibiotics, including carbapenems, cephalosporins, quinolones, and β-lactam/β-lactamase inhibitor combinations. Their MICs for meropenem were high (> 8 mg/L). However, the isolates demonstrated sensitivity to colistin (95.5%), amikacin (53.8%), and trimethoprim/sulfamethoxazole (34.4%).

A comparison between the results from the phenotypic test (CDT) and rtPCR showed high concordance (97.7%), with discrepancies in only two isolates. One isolate was positive by CDT for *OXA-48* but showed no genes detected by rtPCR, while another was positive by CDT for *OXA-48*, but rtPCR identified the *NDM* gene. This indicated that the phenotypic assay could be useful for the early detection of resistance mechanisms, serving as a valuable part of routine susceptibility testing.

Knowledge of mechanisms through phenotypic methods is essential in various clinical scenarios and must be reported within 48 h [26].

In our study of 87 CRKP strains, rtPCR results showed that 85 (97.7%) tested positive for the carbapenem resistance gene. The *OXA-48* gene was predominant, detected in 83 (95.4%) isolates, while *KPC* and *NDM* were each found in one isolate. No *VIM* or *IMP-1* genes were identified, and two isolates were negative on both rtPCR tests.

These findings indicate that *OXA-48* is the principal contributor to *Klebsiella pneumoniae* resistance to carbapenems at our hospital, aligning with previous reports from 2017 and 2018 at the Clinical Center of the University of Sarajevo, Bosnia and Herzegovina, where *bla_OXA-48_* was the most commonly identified carbapenemase gene. The latest data from this study, which involved 87 CRKP isolates observed over one year, demonstrate a notable surge in carbapenem resistance. Potential causes include horizontal transmission and the overutilization of antibiotics, exacerbated by changes implemented in response to the emergence of the new coronavirus, intended to mitigate the spread of SARS-CoV-2. This could also indicate that this strain has become endemic in our hospital [25].

In our study, MLST analysis of ten CRKP isolates revealed that ST101 was the most prevalent, accounting for 90% of cases, while ST307 was detected in only one isolate.

ST101 is a recently identified clone that poses a significant risk due to its high level of multidrug resistance. It is a member of the clonal complex 11, which is associated with various ESBL types, including cefotaximase-Munich 15 (CTX-M-15), OXA-48, NDM-1, and KPC. The carbapenem-resistant ST101 isolates have spread globally and are implicated in healthcare-associated infections across Europe, Asia, the USA, and Latin America [27, 28].

We have identified five types of plasmid replicons across our ten isolates. Col440II was the predominant replicon type, present in 90% of the isolates, followed by IncR at 70%. Both IncFIA(HI1) and repB(R1701) were present in 50% of the isolates. The remaining plasmid replicon, IncFIB(K), was detected in the ST307 isolate, accounting for 10% of the total.

In their 2023 study, Cirkovic et al. [29] reported that the majority of *K. pneumoniae* isolates possessed four or more different plasmid replicon types, with Col440II being the most common, occurring in 75% of the cases. Spadar et al. analyzed plasmid replicons in a large global dataset of 12,468 *K. pneumoniae* isolates and found that the most frequent replicon type was IncFIB(K), present in 3123 (24%) isolates. In contrast, Col440II was detected in only 16 isolates from Italy [30].

In our study, different acquired antimicrobial resistance genes were identified across ten CPKP isolates. All ST101 isolates carried the *bla_CTX-M-15_* gene, and five of these harbored *bla_OXA-48_*. One ST101 isolate (ID 16) contained *bla_OXA-1_* beside the *bla_OXA-48_* gene. No carbapenemase-producing genes were detected in the ST307 isolate.

Phylogenetic analysis revealed epidemiological links among certain isolates. With the increasing affordability of next-generation sequencing (NGS), its application in hospital settings is proving highly advantageous, enabling the use of acquired data to guide strategies for infection control and prevention.

The phylogenetic analysis of the ten CRKP isolates identified three clusters of ST101 that could constitute an outbreak, given a cutoff of ten SNPs. CRKP strains are highly transmissible within hospital settings, spreading from patient to patient either through direct contact with healthcare workers or indirectly through the environment. However, the gastrointestinal tracts of colonized patients may serve as the main source for hospital outbreaks. Consequently, the utilization of rectal surveillance cultures to actively screen for colonization, combined with the implementation of contact precautions, has proven very effective in reducing the transmission of CRKP among patients [24, 25, 31–33].

Our study has several limitations that should be considered. The study was conducted at a tertiary hospital in Sarajevo; hence, the findings may not be generalizable to other institutions. To gain a broader understanding of the clinical significance of CRKP strains in our nation, it is necessary to collect isolates from multiple centers. Additionally, the absence of detailed clinical data, such as records of previous hospitalizations or antibiotic treatments, limits our ability to identify patients at high risk of carrying CRKP.

## Conclusion

The findings from this study provide valuable insights into the genetic characteristics of CRKP isolates circulating in a Sarajevo tertiary hospital during 2022. *OXA-48* emerged as the predominant carbapenemase gene, with ST101 being the major MLST type detected. Given the significant resistance demonstrated by the CRKP isolates to most tested antibiotics, it is imperative to establish effective methods to restrict the spread of these isolates, as well as to carefully monitor the use of carbapenems in clinical practice.

## Data Availability

The data presented in this study are available from the corresponding author upon reasonable request.
